# Neurophysiological correlates of interpersonal discrepancy and social adjustment in an interactive decision-making task in dyads

**DOI:** 10.3389/fpsyg.2024.1272841

**Published:** 2024-02-14

**Authors:** Unai Vicente, Alberto Ara, María Palacín-Lois, Josep Marco-Pallarés

**Affiliations:** ^1^Department of Cognition, Development and Educational Psychology, Institute of Neurosciences, University of Barcelona, Barcelona, Spain; ^2^Department of Social and Quantitative Psychology, University of Barcelona, Barcelona, Spain; ^3^Cognition and Brain Plasticity Unit, Bellvitge Biomedical Research Institute, Barcelona, Spain; ^4^Cognitive Neuroscience Unit, Montreal Neurological Institute, McGill University, Montreal, QC, Canada; ^5^BRAMS: International Laboratory for Brain, Music and Sound Research, Montreal, QC, Canada

**Keywords:** ERPs, EEG, dyadic decision-making, social cognition, adjustment

## Abstract

**Introduction:**

The pursuit of convergence and the social behavioral adjustment of conformity are fundamental cooperative behaviors that help people adjust their mental frameworks to reach a common goal. However, while social psychology has extensively studied conformity by its influence context, there is still plenty to investigate about the neural cognitive mechanisms involved in this behavior.

**Methods:**

We proposed a paradigm with two phases, a pre-activation phase to enhance cooperative tendencies and, later, a social decision-making phase in which dyads had to make a perceptual estimation in three consecutive trials and could converge in their decisions without an explicit request or reward to do so. In Study 1, 80 participants were divided in two conditions. In one condition participants did the pre-activation phase alone, while in the other condition the two participants did it with their partners and could interact freely. In Study 2, we registered the electroencephalographical (EEG) activity of 36 participants in the social decision-making phase.

**Results:**

Study 1 showed behavioral evidence of higher spontaneous convergence in participants who interacted in the pre-activation phase. Event related Potentials (ERP) recorded in Study 2 revealed signal differences in response divergence in different time intervals. Time-frequency analysis showed theta, alpha, and beta evidence related to cognitive control, attention, and reward processing associated with social convergence.

**Discussion:**

Current results support the spontaneous convergence of behavior in dyads, with increased behavioral adjustment in those participants who have previously cooperated. In addition, neurophysiological components were associated with discrepancy levels between participants, and supported the validity of the experimental paradigm to study spontaneous social behavioral adaptation in experimental settings.

## 1 Introduction

One of the critical mechanisms involved in human cooperation is influence. Seminal social psychology experiments showed that people tend to match each other’s behavior (Asch, 1956) and perception (Sherif, 1935, 1958) as part of an automatic psychosocial mechanism. Both Asch’s and Sherif’s interpretations might fall reduced, as they assume social influence is based on norm deviation aversion or precision-seeking, respectively. Deutsch and Gerard (1955) were the first to point out this compatibility of visions, who distinctly identified normative conformity (Asch’s view) and informative conformity (Sherif’s view). This vision has also configured today’s more modern takes on conformity (Kendal et al., 2018), explaining it as a social behavioral adjustment sourced in a foraging information system inside social decision-making based on diverse influences (Campbell-Meiklejohn et al., 2010; Toelch and Dolan, 2015) and sourced on a particular regime of expectations (Constant et al., 2019). Therefore, as a mechanism that promotes and facilitates cooperation, conformity is a type of prosocial behavior that happens when a person changes their judgments and decisions to match those of another person or group. Conformity is crucial in cooperation and involves adjusting one’s view or behavior in favor of a shared framework with others to reach a synergic goal (despite their interests). Interestingly, people tend to adjust their behavior to converge to others’ even without explicit instruction or reward. This adaptation seems almost automatic and has been demonstrated as a powerful force in shifting people’s decision-making, even by positive contagion (Nook et al., 2016). However, despite the extensive neuroscience literature on different social interactions, studies on the neural correlates of social conformity using simultaneous decision-making and its associated behavioral adaptation mechanisms have been scarce and, in fact, few studies have examined the brain mechanisms of behavioral adaptation due to social factors. However, most studies have focused on behavioral adaptation caused by other sources, such as changes in rewards (e.g., changes in the action-reward contingencies, Mas-Herrero and Marco-Pallarés, 2014) or the agent’s internal states (Cavanagh et al., 2011). Under the active inference framework, Bayesian principles driving attention and perception constantly reshape our expectations, consequently guiding our actions (Constant et al., 2019). Therefore, the brain mechanisms responsible for the adjustments performed in social conformity situations could be similar to those involved in behavior adjustment due to environmental contingencies. Accordingly, adjusting behavior in the face of external signals, social or otherwise, would require, at least, two steps: first, detecting a discrepancy between the performed actions and other sources of information, and second, allocating the necessary cognitive resources to take the appropriate measures to correct or adapt this response.

Detecting discrepancies with previous stimuli has been traditionally related to different electrophysiological brain responses. Although there is still an ongoing debate on the model that best represents the conflict monitoring computational schema in the brain (Holroyd et al., 2008; Shenhav et al., 2013, 2016; Vassena et al., 2017, 2020), there is a consensus that these error systems are driven by prediction errors (Holroyd and Coles, 2002). Previous studies have reported that a negative frontocentral deflection appears after negative feedback (FB), peaking 250-300 ms after FB onset, the so-called Feedback Related Negativity (FRN; Miltner et al., 1997). This event-related potential is sensitive to the magnitude of the loss and the likelihood of the negative FB and has been proposed to be related to reward prediction errors (Sambrook and Goslin, 2015). Interestingly, this subcomponent of ERP signal has already been associated with social conformity based on subjective norm-related measures such as attractiveness rating (Shestakova et al., 2013; Schnuerch et al., 2015) or informational foraging paradigms (Wang et al., 2020). However, even though these might simulate conditions of conformity, behavioral adaptation is induced, and its sociality is assumed *de facto*. Another essential component related to behavior adjustment is the P300 ERP (Polich, 2003, 2007). Several studies have consistently reported that the P3 ERP is associated with attentional changes needed to allocate attention to relevant changes in the environment and the required targets (Polich, 2003, 2007). The P3 has traditionally been divided into two main components. P3a is related to attentional processes driven by context (Katayama and Polich, 1998) or emotional value (Delplanque et al., 2006), among many others. This component has been associated with behavioral adjustments and switching (Polich, 2003, 2007). The P3b subcomponent, on the other hand, is related to cognitive engagement operations and a memory-storage mechanism coming after such engagement (Kropotov, 2010). Higher P3b amplitudes are related to target identification in the working memory updating process (Rac-Lubashevsky and Kessler, 2019) which might be relevant in higher-level adjustments associated with social convergence. Furthermore, in a recent study, researchers related centroparietal positivity (CPP) and late positivity (LP) elicited by outcome feedback as a predictor of subjects’ change in their responses (Bogdan et al., 2022). In addition, decision-making studies have also revealed a crucial role of theta oscillatory activity in cognitive control (Cavanagh et al., 2010; Cavanagh and Frank, 2014), conflict (see Polich (2007) for a review), and computation of surprises or prediction errors (Alexander and Brown, 2011; Cavanagh et al., 2012; Mas-Herrero and Marco-Pallarés, 2014). More importantly, recent literature relates theta oscillatory activity to outcome evaluation influenced by others’ opinions (Wang et al., 2020). In summary, we would expect that in a social behavioral adaptation scenario, the discrepancy between one’s and partners’ responses would be indexed by an increase in the FRN and the theta oscillatory activity. In contrast, the attentional demands and adjustments associated with the operations needed to adapt or change the response would be reflected by an increase in the P3 ERP. In addition, other frequency components have been found to be involved in decision-making. In concrete, alpha oscillatory activity indexes inhibitory processing and its reduction is linked to the disinhibition of the brain areas required in a cognitive function. In this sense, enhanced alpha desynchronization is related to increase in the attentional demands in a task and related to better performance (Glazer et al., 2018). In addition, previous studies have described an increase in the beta oscillations after positive feedbacks (Marco-Pallarés et al., 2008; Mas-Herrero et al., 2015), especially in those unexpected or highly relevant rewards (Marco-Pallarés et al., 2015). Different accounts have been proposed for the function of such response including a role in the prolongation of current cognitive state (Engel and Fries, 2010), a fast motivational value signal (Marco-Pallarés et al., 2015) or a response associated with flexible cognitive control (Lundqvist et al., 2018).

Prosocial behaviors have been traditionally studied in neuroscience using simulated social paradigms rather than actual social interactions, despite the required neural processes being highly dependent on the agent’s immediate altered by mutual affective and cognitive influence (see Stallen and Sanfey, 2015 for a review). However, the domain of cooperative decision-making has been dominated by experimental paradigms inspired by the game theory, such as the prisoner’s dilemma (see Liu et al., 2018; Redcay and Schilbach, 2019 for a review), which usually simplifies cooperation as being contrary to competition. However, recently this norm has begun to change. In a recent study, researchers alternated the roles of responder and proposer in an ultimatum game to induce conformity by repetition (Bogdan et al., 2022). This study is the first of its kind using a dual-person paradigm where the decisions are biased by recent interactive history. Although the results in this experiment serve as a reference point, we wanted to explore further the conformity phenomena in a synchronous interactive paradigm. Other studies on conformity also rely on paradigms that simulate group pressure on a single person’s decision-making. In these paradigms, the behavioral adaptation is only triggered by reviews or opinions from a distant and unknown group (Klucharev et al., 2009; Campbell-Meiklejohn et al., 2010; Zaki et al., 2011; Morgan et al., 2012; Shestakova et al., 2013; Schnuerch et al., 2015; Xie et al., 2016; Liuzza et al., 2019; Overgaauw et al., 2019; Li et al., 2020; Wang et al., 2020; Duell et al., 2021).

The present experiment aimed to study the mechanisms behind willful convergence in a fully cooperative decision-making task. First, we wanted test whether participants would spontaneously converge in a task and if previous cooperation would enhance such convergence. We designed a new experimental paradigm with two parts to reach this goal. First, we conducted a behavioral study (Study 1) to test the new paradigm. We tested 80 participants separated into two randomized groups, where half of the participants were assigned to the “Cooperation” group and the others to the “Individual” group. The only difference in the paradigm was how they performed the pre-activation task if they did it alone or with their partners. In the second phase of the paradigm, the two participants had to simultaneously determine a point’s position on the screen inspired by the norm-related seminal study in social psychology known as the autokinetic effect by Sherif (1936). They had three attempts for each decision and were informed about their partner’s response after each decision. Even if not explicitly stated/instructed, we hypothesized that participants would tend to converge in their responses. In addition, we aimed to study the neurophysiological correlates of social conformity when participants were informed about the decision of their partners (Study 2).

We hypothesized that the brain responses, which have traditionally been related to discrepancy (theta activity) and attentional demands (FRN, P3, and LP), would be associated with the automatic adaptation of behavior in this social paradigm and would be modulated by the degree of adaptation and change performed by participants. Additionally, we are analyzing all data from a single-trial perspective that facilitates the study of other cognitive process differences associated with participant’s responses (i.e., their intra-personal or inter-personal adjustments, pre-conformity activity) as well as role-related differences regarding their level of conformity in the trial.

## 2 Materials and methods

### 2.1 Participants

80 psychology students (40 randomly assigned dyads) from the University of Barcelona participated in Study 1. They were also randomly assigned to two different groups. Participants signed informed consent before the experiment and received a point-based reward for their grades. All sessions were recorded in audio and video under all participants’ consent. Our sample consisted of 63 females and 17 males (30 females and 10 males, aged: 18–48, in the “Cooperative” group; 33 females and 7 males, aged: 18–58, in the “Individual” group). The participants were randomly paired into dyads, with the only limitation being that they did not know each other beforehand.

In Study 2, 44 participants (24 females and 20 males, aged: 19–58), different from Study 1, were randomly assigned to pairs (dyads), with the only criterion being not knowing each other before the experiment. Four dyads were excluded from the experiment due to technical problems, resulting in a final sample of 18 dyads (36 participants: 20 female and 16 male, Age Median: 24, range: 19–53). All participants signed informed consent before participating and received a monetary payment of €30 for participating in the experiment. The experiment took an average of 3 hours.

The Bioethical Commission of the University of Barcelona approved the experiment.

### 2.2 Phase 1: pre-activation task

**Figure 1:**
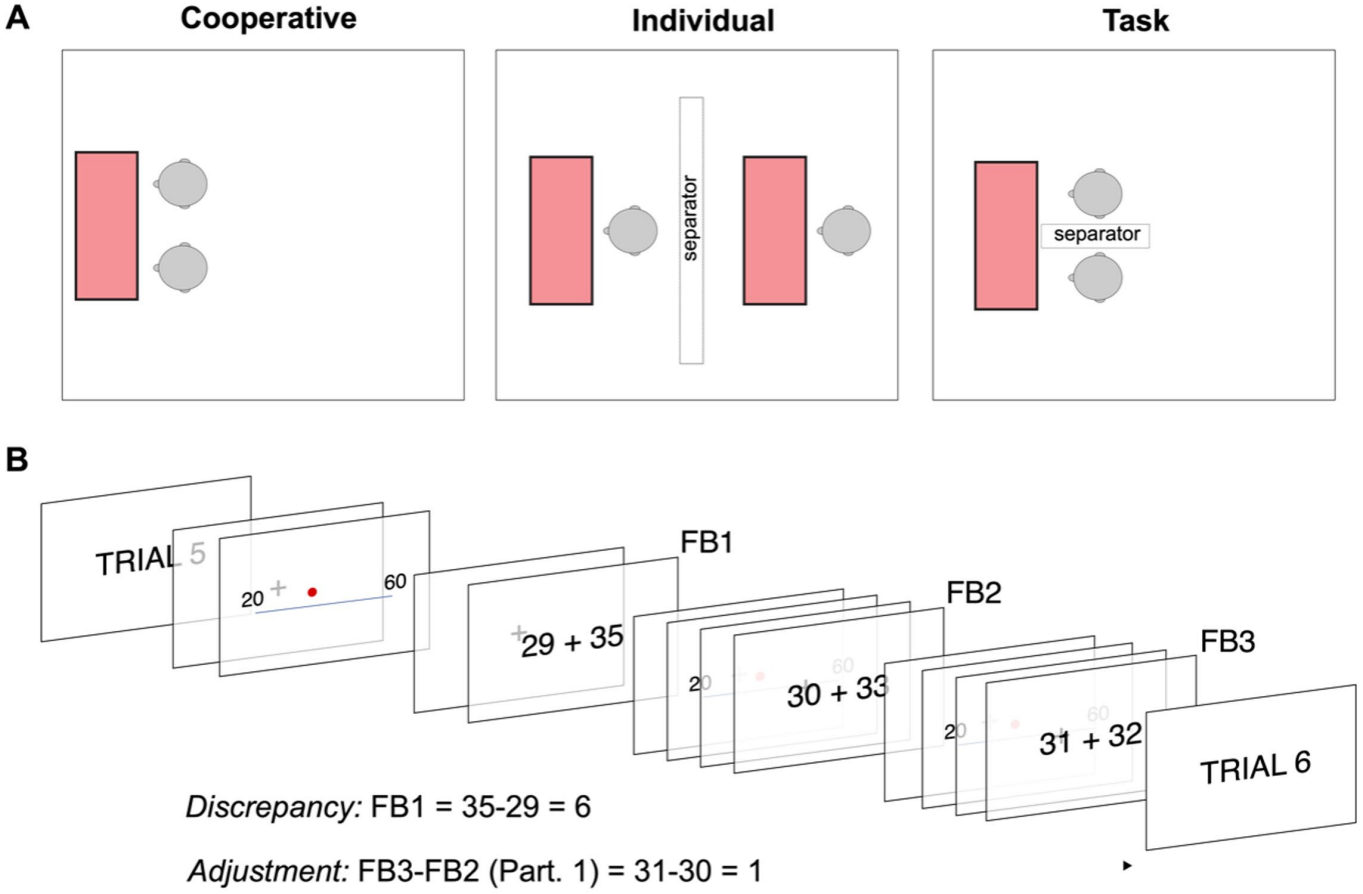
**(A)** Disposition of the lab for the different group configurations in Study 1. Left: Cooperative dyad setting; Middle: Individual dyad setting; Right: during the task. Note that in Study 2, all participants were in the Cooperative setting. **(B)** Design of the task. The trial started with its number, and then a red point appeared on a line between two numbers. Participants had to enter the estimation of the position of the point using individual keypads, and after the two participants had pressed the intro key, the estimation of the two participants appeared on the screen (FB1). Then the same sequence with the same figure was presented two more times, and participants received the information about their and their peers’ estimation (FB2 and FB3). After that, the new trial started. The figure also explains what “discrepancy” and “adjustment” mean in our experiment.

The experiment consisted of two main parts. In Study 1, dyads were randomly assigned into two different groups. These groups were named “Individual” (I) and “Cooperative” (C). In Study 2 all participants were assigned to the Cooperative group. All participants were required to complete the same set of tasks with the only difference that if the dyad was in the C group, they were sitting at a table next to each other and they could communicate and interact freely (Figure 1A) to maximize their cooperative interaction (Sommer, 1959) while if they were assigned to the I group the space was separated so, they could not see or interact between them while solving the exercises. The set of tasks was inspired by the cooperative dimension of the circumplex model (McGrath, 1984) and tried to emulate different kinds of tasks that are normally performed in groups to re-create a task-oriented group experience that might lead to a pre-activation of cooperation for the C dyads. All participants had a maximum of 60 min to solve the pre-task, and they were instructed to move forward if they could not solve it in the estimated completion time (Table 1).

**Table 1:** Pre-task design structure, type of task and estimated time of completion.

Type of assignment	Type of task	ETC	Description
Estimation questionnaire	Compensatory/discretionary task	10′	Eight different questions that require association of different aspects of general knowledge (e.g., the average weight of a hippopotamus)
Puzzle solving	Conjunctive task	20′	Solve manipulative tasks such as a tangram figure and building the biggest vertical tower possible.
Team profiles	Decision-making task	7′	With a description of 6 different profiles, decide their roles in a team with a particular mission
Logo creation	Creativity task	7′	Design a logo for a local grocery shop
Faces: judgement	Categorization task	7′	Sort a set of different faces, starting with those who are considered to depict the happiest emotional reaction to those who seem sadder
Estimating time	Time synchronization task	4′	Try to estimate (looking each other in the eyes in the C group) when a certain period passes (37 s, 1 min, 1:45 s)
Imitation of postures and faces	Joint action task	5′	Imitate the body figures in six photographs of people practicing different yoga positions (imitate simultaneously in the C group)

Participants had a maximum of 60′ to complete the entire answer sheet.

ETC, estimated time of completion.

After the pre-task, participants started the second phase of the experiment following the same disposition in the room. Distinctly, in Study 2, an EEG headcap was mounted onto each participant. Participants sat in a comfortable chair and responded via a numeric keypad. In Study 2, before the beginning of the task, participants were asked to relax for 3 min by listening to a pre-recorded guided relaxation. The reason behind this is after the pre-activation task participants had to wait until the EEG headset was successfully installed which might have taken them off the experiment. We intentionally wanted all participants to start from the most comparable psychological condition possible. After that, two training trials were presented, and the main task started.

### 2.3 Phase 2: task

The task consisted of 160 trials in Study 1 and 100 trials in Study 2. In each trial, a vertical or horizontal line appeared on the screen, with two numbers at each end indicating the arbitrary limits of the line. The numbers were randomly selected between 0 and 150 and within a range between 40 and 50 units. In addition, a red point appeared at a random position along the line. Participants were required to write the position they estimated for this point as a number. After the two users had introduced their inputs and pressed the intro button, a cross was displayed on the center of the screen for 0.5 s. Then participants saw the two inputs (own and partner’s), so they could evaluate the difference and adjust, or not, the estimation. After this, the next repetition in the same trial started when the two participants pressed the intro button. The same line and red dot were presented three consecutive times so that participants could change their estimation at their will. Onwards, we will identify these FB as first feedback (FB1), second feedback (FB2), and third feedback (FB3). However, and very importantly, participants were neither explicitly nor implicitly encouraged or rewarded to coincide in their estimations. Presentations of the stimuli were the same in both studies, as seen in Figure 1, although the feedback presentation changed in Study 2, where we added a fixation point prior to the FB (Figure 1B).

The main task was programmed using Python 2.7. Study 1 divided the task into four blocks of 40 trials per block with three repetitions of the same stimuli per trial. Study 2 divided the experiment into four blocks of 25 trials per block with three repetitions of the same stimuli per trial. At the end of every block, participants could rest before moving forward to the next block (both participants had to press their enter button) whenever they were ready. They were instructed to avoid speaking or communicating between themselves in any way (e.g., giggling, sighing) during the task.

### 2.4 Post-task survey

In Study 1, after the task, every participant completed a custom-made survey requesting information about the general perception of the experience of the experiment. Four questions were asked to measure the perceptive and subjective experience regarding likeability, synchronicity, trust, and reward. The questions were A) “Did you like the experiment?” which we have called “Likeability” B) “Did you feel synched with your partner?” which we called “Synchronicity,” C) “Did you find you could trust your partner?,” which was called “Trust” and D) “Did you find rewarding working with your partner?” which was called “Reward.” They had a Likert type scale, starting from 1 (lower) to 5 (higher).

### 2.5 EEG processing

In Study 2, EEG was recorded using an ANT Neuro ASALab EEG amplifier at 1024 Hz using two different elastic caps from 27 scalp electrodes (Fp1/2, Fz, F3/4, F7/8, FC1/2, FC5/6, Cz, C3/4, Cp1/2, CP5/6, Pz, P3/4, P7/8, Poz, Oz, M1/2). Eye movements were registered with an electrode associated with the participant’s dominant hand at the infraorbital ridge of the eye. The electrode impedance was kept below 5kΩ during the task.

The electrophysiological signal was bandpass filtered, with cut-off frequencies of 0.1 Hz to 30 Hz using nose as physical reference and re-referenced offline to the activity of the two mastoids. Epoch events were extracted from −2 to 2 s after the stimuli showing the estimated position of each participant (feedback), using a baseline from −100 ms to 0 ms. Independent Component Analysis (ICA) (Makeig and Onton, 2011) was used to clean artifacts and, afterwards, epochs exceeding ±100 μV from -100 ms to 1,000 ms were automatically rejected. Time-frequency (TF) analysis was also computed by convolving single trials with 7 cycle complex Morlet wavelet for frequencies ranging from 1 to 30 Hz. Changes in power were computed by dividing power value by baseline (−400 ms to −100 ms) for each electrode, frequency, and condition.

The ERP/TF analysis focused on three midline electrodes (Fz, Cz and Pz) and the different time ranges were based on visual inspection of the corresponding ERPs. The topography of the studied components justified the use of these electrodes. Statistical analysis of time-frequency data was performed in theta (4-8 Hz), alpha (8-12 Hz) and beta (12-30 Hz) bands.

### 2.6 Bayesian multilevel modelling

We used Bayesian Multilevel Modelling (BMM) using all the available data by trial due to its stability (Baayen et al., 2008) in experimental designs with repeated measures and multiple comparisons (Ara and Marco-Pallarés, 2020). The main advantage of this method, among others, is its high performance when working with small sample sizes. Using all the available data avoids the need for asymptotic approximations and provides high interpretability (Baayen et al., 2008; Keysers et al., 2020). BMM gives an objective alternative to frequentist corrections in multiple comparisons (Berry and Hochberg, 1999; Gelman et al., 2012), using priors centered at 0, and informing custom hierarchical priors when a hierarchical model requires it, making Bayesian inference highly conservative (Gelman et al., 2012). Separate intercepts and slopes were used for each dyad. To define the random slope model, we defined a nested random term (participants nested in dyads) defined by our experimental design. In other words, by defining this factor in the random term, we let our model calculate co-dependent intercepts by dyad. We used the same model structure for behavioral data, ERP, and time-frequency analysis.

Posterior samples were computed using the outcome of 4 independent chains initialized at 0, and all the partial variabilities were added according to the model. After modeling, inferences were computed using the Highest Density Interval (HDI) of 95% (Kruschke, 2014) to check the inclusion of the null hypothesis in the posterior models, and hypotheses were tested as proposed in Kruschke (2018) and Kruschke and Liddell (2018). In addition, we used, as suggested by Kruschke (2018), a decision rule considering, together with the HDI, a region of practical equivalence (ROPE) around the null value. The ROPE range was adjusted to every contrast by multiplying the variability, SD*y*, by ±0.05, so we had an approximate, highly conservative, ±0.05*SD*y* ROPE range. In time-frequency analysis, because reductions in power data are on a much lower scale than voltage data, and so is its variability, we decided to reduce the ROPE range to ±0.01*SD*y*. Accordingly, we rounded to two decimals of the HDI in the behavioral and ERP results and three in the Time-Frequency HDIs. It is also important to note that we considered behavioral interpersonal distance a *Hurdle-Gamma* distribution by fitting different distributions and using the leave-one-out (loo; Vehtari et al., 2017) technique. EEG signal models followed a *student’s t* distribution, while time-frequency power models followed a *Gamma* ( γ ) distribution with a log link. We chose this link function to the γ because, contrary to the canonical link, the log link produces a multiplicative model on the original scale, which allows a straightforward interpretation of its results. We reported SD*y* maximum (SD*y*_max_) and minimum (SD*y*_min_) limits from likelihood distribution (data) after reporting the results of every model.

Furthermore, we reported as credible only the results within the HDI + ROPE decision rule, where the entire HDI fell outside the ROPE. Posterior distributions were computed with four Markov chains initialized at zero with 10,000 samples. The first 1,000 were discarded as warmup (we doubled the iterations and increased warmup for specific models to reduce divergent transitions). The target acceptance rate or parameter “adapt_delta” was set to 0.9 (increased to 0.99 for the discrepancy in the FB1 model) according to the type of parameters for sampling algorithms. The No-U-turn sampler (NUTS) algorithm maximum tree-depth parameter was set to 10 in all the models to maximize the depth of the trees at each iteration (Bürkner, 2017). All models converged with those parameters according to split-R-hat criteria (Gelman et al., 2013). Our models were built based on the intercept and subparts (differences) extracted from posteriors. Interpersonal divergence in behavioral analyses and voltage (or power in the case of time-frequency) was the dependent variable in our model (*y*), and the rest of the measures were predictors, alone or in interaction.

### 2.7 Model specification

Table 2 is a summary of all models used. In all the interaction models, we used hierarchical priors suggested by Gelman and Hill (2006) for higher consistency. In Study 1, we modeled discrepancy (difference between the estimation provided by the two participants) predicted by the interaction with group (G) condition and FB (*discrepancy_ijk_ ~ β*_0*ij*_ **+** *β*_1*ij*_x*_FBk_* **+** *β*_2*ij*_x*_Groupk_* **+** *β*_3*ij*_ x*_FB_* x*_Groupk_* **+** *ε_ijk,_* being *k* the predicted measure of the *j*-th participant in the *i*-th dyad). We removed outliers (P95), clearly unintentional typos, and replaced them with a missing value (NA) as they are automatically removed from the model. Additionally, in Study 2, we also model the discrepancy to the FB (*discrepancy_ijk_ ~ β*_0*ij*_ **+** *β*_1*ij*_x*_FBk_* **+** *ε_ijk_*). In both models, we used a zero-inflated *hurdle-gamma* distribution family to model the discrepancy.

**Table 2:** Summary of all the models used in this article.

Model ID	Type (Study)	FB	Variable	Notation	R Notation
Group Interaction	Behavioral (S1)	All	FB, Group	*discrepancy_ijk_ ~ β*_0_ **+** *β*_1_x*_FBijk_* **+** *β*_2_x*_Gijk_* **+** *β*_3_ x*_FB_* x*_Gijk_* **+** *ε_ijk_*	Discrepancy~Feedback*Group + (1|ID/Suj)
Feedback Simple	Behavioral (S2)	All	FB	*discrepancy_ijk_ ~ β*_0_ **+** *β*_1_x*_FBijk_* **+** *ε_FB|ijk_*	Discrepancy~Feedback + (Feedback|ID/Suj)
Accuracy	Behavioral (S1)	All	Accuracy, Group	*accuracy_ijk_ ~ β*_0_ **+** *β*_1_x*_Gijk_* **+** *ε_ijk_*	Accuracy~Group + (1|ID/Suj)
Simple Question Models	Behavioral (S1)	All	Answer, Group	*answer_i_ ~ β*_0_ **+** *β*_1*i*_x*_G_* **+** *ε_i_*	Answer~Group
Feedback Signal	ERP/TF (S2)	All	FB	*signal_ijkt_ ~ β*_0*t*_ **+** *β*_1*t*_x*_FBijk_* **+** *ε_ijk_* **+** *ε_FB||t_*	Signal~Feedback + (1|ID/Suj) + (Feedback||Time_Electrode)
Discrepancy FB interaction	ERP/TF (S2)	All	Discrepancy, FB	*signal_ijkt_ ~ β*_0*t*_ **+** *β*_1*t*_x*_FBijk_* **+** *β*_2*t*_x*_Dijk_* **+** *β*_3*t*_ x*_FB_* x*_Dijk_* **+** *ε_ijk_* **+** *ε _FB_* × *_D||t_*	Signal~Feedback*Discrepancy + (1|ID/Suj) + (Feedback*Discrepancy||Time_Electrode)
Adjustment FB interaction	ERP/TF (S2)	FB2, FB3	Adjustment, FB	*signal_ijkt_ ~ β*_0*t*_ **+** *β*_1*t*_x*_FBijk_* **+** *β*_2*t*_x*_Aijk_* **+** *β*_3*t*_ x*_FB_* x*_Aijk_* **+** *ε_ijk_* **+** *ε _FB_* × *_A||t_*	Signal~Feedback*Adjustment + (1|ID/Suj) + (Feedback*Adjustment||Time_Electrode)

“Model ID” identifies the model; “Type/Study” explains where the model has been used; S refers to study.

S1 and S2 = study 1 and study 2.

In the analysis of the post-experiment questionnaires in Study 1 responses were treated as the dependent variable. They followed a cumulative distribution function with *probit* link that predicted the probability of an event occurring, assuming the error was normally distributed. We modeled them with non-informative priors using the group as a predictor. Finally, in Study 1, we also compared the difference between the responses given by the participants with the real position of the point to determine whether there were differences between the two groups. We took the centered difference at the last repetition of each trial between both participants and compared it to the actual point getting an accuracy value per trial that we later modeled as an independent variable and related to group (*accuracy_ijk_ ~ β*_0*ij*_ **+** *β*_1*ij*_x*_Groupk_* **+** *ε_ijk_*).

In the ERP models of Study 2, we used four different indexes, being *k* the signal measure of the *j*-th participant in the *i*-th dyad, at uncorrelated random effects slope for the last index, time-range/electrode (*t*). First, we analyzed the effect of feedback presentations in the different ERP and time-frequency components (*signal_ijkt_ ~ β*_0*ijt*_ **+** *β*_1*ijt*_x*_FBk_* **+** *ε_ijk_* **+** *ε_FB||t_*). Then, we aimed to study in a trial-by-trial basis how the discrepancy (D, difference in the responses of the two participants of the dyads) and adjustment (A, change in the response estimation after observing other participant’s response) were associated with the electrophysiological responses. We created a model aiming to explain how changes in the electrophysiological activity between feedbacks were explained by changes in the discrepancy at different feedback presentations (*signal_ijkt_ ~ β*_0*t*_ **+** *β*_1*t*_x*_FBijk_* **+** *β*_2*t*_x*_Dijk_* **+** *β*_3*t*_ x*_FB_* x*_Dijk_* **+** *ε_ijk_* **+** *ε _FB_* × *_D||t_*). In this latter case, changes in discrepancies were min-max scaled concerning the discrepancy of the first feedback. A similar approach was used in the study of the adjustment, in which the changes in the different ERPs and time-frequency components were explained by the scaled adjustment of the participant (*signal_ijkt_ ~ β*_0*t*_ **+** *β*_1*t*_x*_FBijk_* **+** *β*_2*t*_x*_Aijk_* **+** *β*_3*t*_ x*_FB_* x*_Aijk_* **+** *ε_ijk_* **+** *ε_A||t_*). Because the adjustment was made after processing the feedback, we modelled the signal in one trial with the adjustment visible in the next, hypothesizing we could capture signal related to the response evaluation process. Importantly, we removed the trials where both participants reached convergence at first FB in both models as it did not provide any new information to the model but inserted noise.

Weakly informative priors were used for the intercept and slope (normal, μ = 0, σ = 1) and for the varying effects (gamma, α = 1, β = 10) for the simplest feedback repetition model, and a hierarchical prior in every other interaction model. Outliers (percentile 95) were removed from the model. We showed result differences between maximum (*β*_0_ + *β*_1_*x*; *x* = 1) and minimum discrepancy (*β*_0_; *x* = 0) to let the sign reflect the appropriate reduction (−) or augment (+) in voltage or power. Similarly, we intentionally permuted the contrasts between FB (FB1-FB2 → FB2-FB1) to match the sign of the HDI to the reduction (−) or augment (+) in voltage (or power) at FB and have a more straightforward interpretation of results.

## 3 Results

### 3.1 Behavioral results

**Figure 2:**
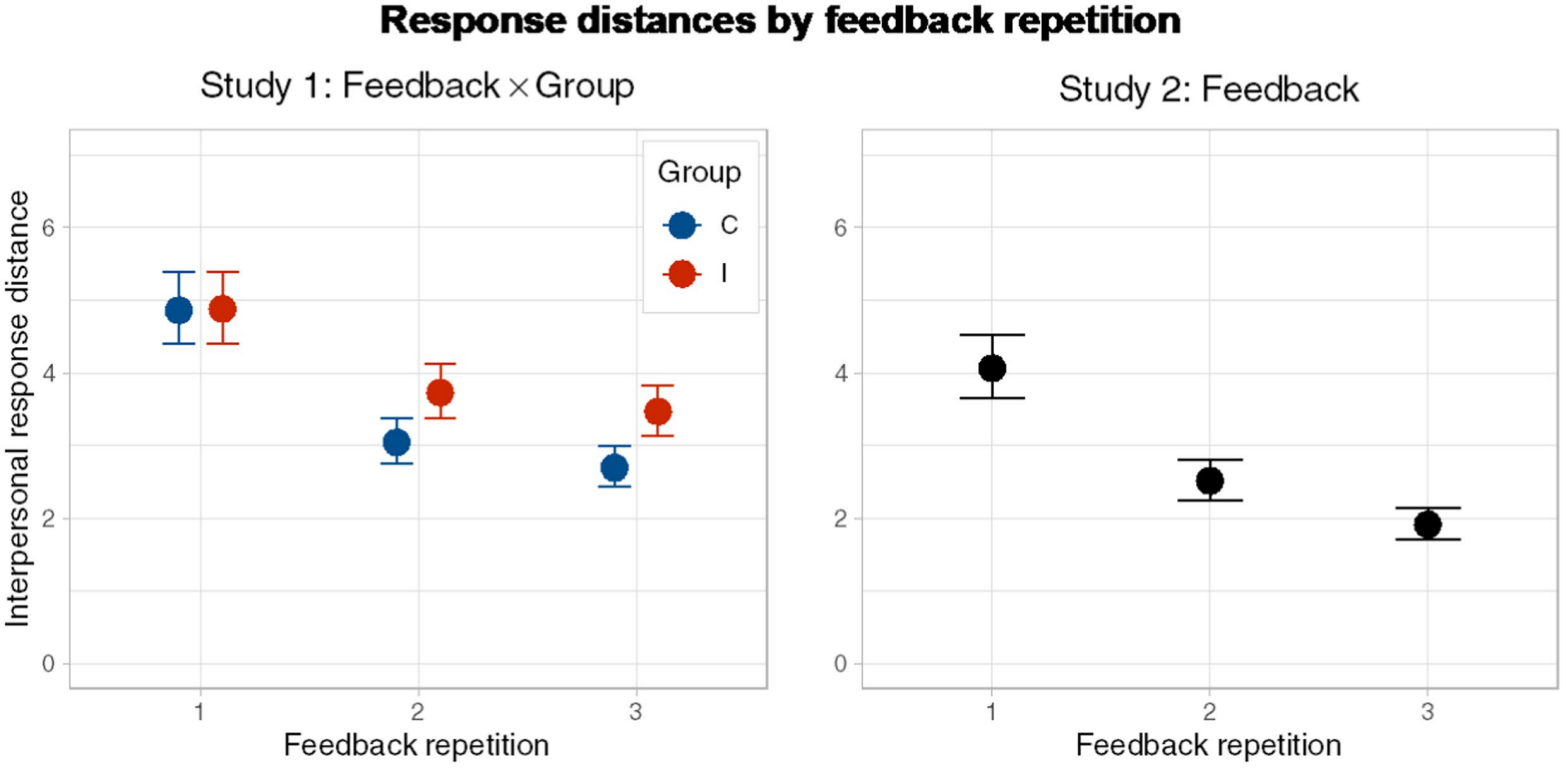
Divergence in responses at every feedback repetition. First plot shows data from Study 1, and also compares between groups. Second plot shows the same tendency in data from Study 2.

Figure 2 shows the interpersonal response distance between both groups. Study 1 showed that regardless of the group, there was a clear tendency to converge as FB advances in both groups. Accordingly, the main effects in our model for Study 1 showed this decrease in the first adjustment (FB2-FB1; HDI(95%): [−0.50 – −0.44]) and also in the second adjustment (FB3-FB2; HDI(95%): [−0.16 – −0.09]), which was replicated in the model from Study 2 (FB2-FB1; HDI (95%): [−0.53 – -0.43]; FB3-FB2; HDI (95%): [−0.33 – −0.21], see Figure 2). These results are evidence of people’s natural tendency to converge when they work together, regardless of their history of cooperation. Nevertheless, considering the differences between groups in Study 1 at every FB, we observed credible evidence of a difference in FB3 (HDI (95%): C vs. I, FB3: [0.10–0.39]) showing cooperation in the pre-task enhanced final inter-personal convergence (higher credible distance at the end of the last trial in the group I compared with the group C. In contrasts, there were not credible evidence on differences in the accuracy of results between groups in Study 1.

**Figure 3:**
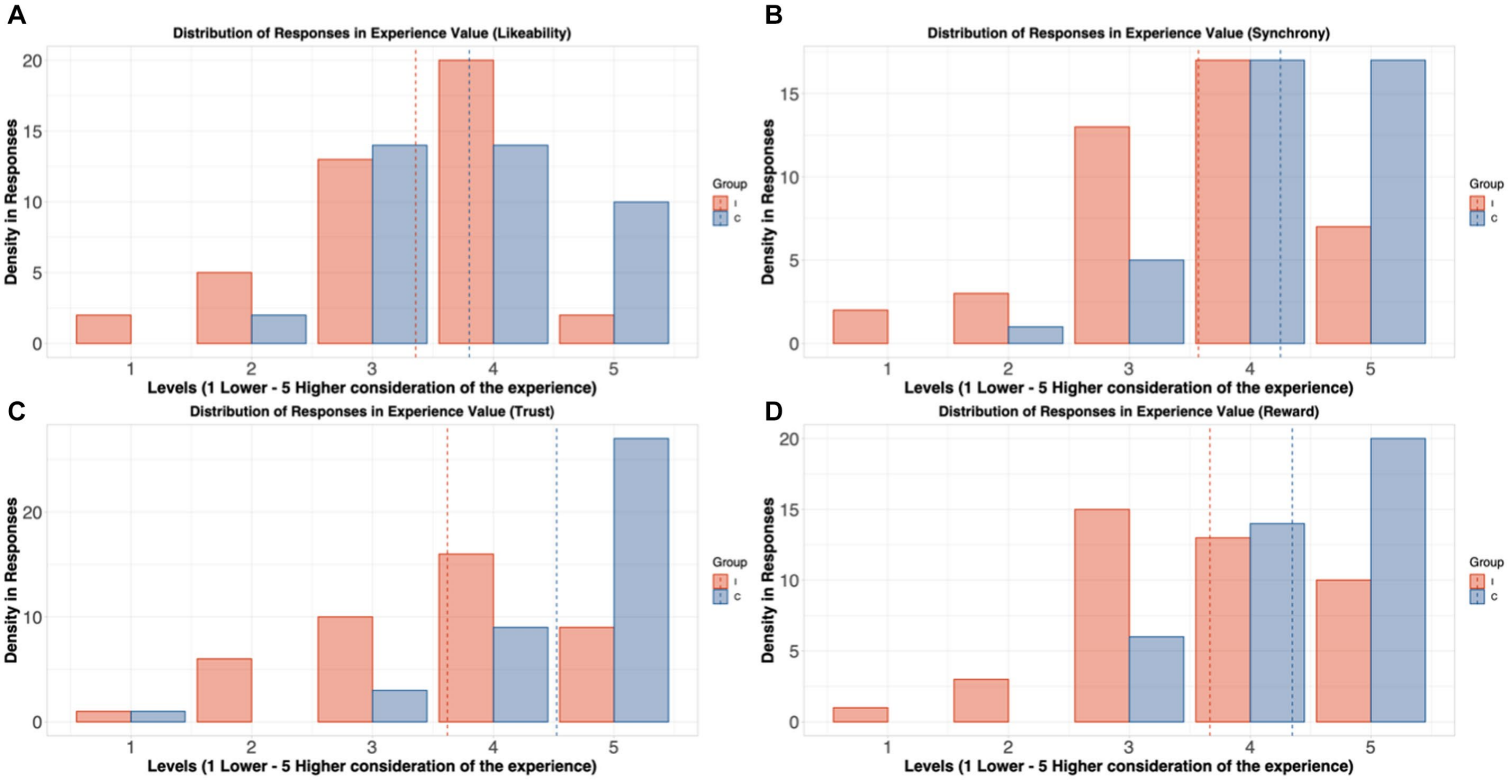
Plots that depict the differences in responses to questions by Group: **(A)** “Did you like the experiment?,” **(B)** “Did you feel synched with your partner?,” **(C)** “Did you find you could trust your partner?” and **(D)** “Did you find rewarding working with your partner?

In the post-experiment survey, we found credibly more negative considerations in all the questions in the group I compared to group C, especially in the last three. In the “likeability” question, even when responses in group I were lower (Figure 3A; “Did you enjoy the experiment?”; HDI (95%): [−1.01 – −0.07]), evidence was not credibly strong to reject within ROPE. Nonetheless, we report credible solid evidence of a diminished consideration in responses from “synchronicity” question (Figure 3B; “Did you feel synched with your partner?”; HDI (95%): [−1.32 – −0.34], “trust” related question (Figure 3C; “Did you feel you could trust your partner?”; HDI (95%): [−1.63 – −0.60] and, finally, the question about “reward” (Figure 3D; “Did you find rewarding working with your partner?”; HDI (95%): [−1.34 – −0.36] in group I compared to responses in group C.

### 3.2 ERP results

#### 3.2.1 Feedback model

**Figure 4:**
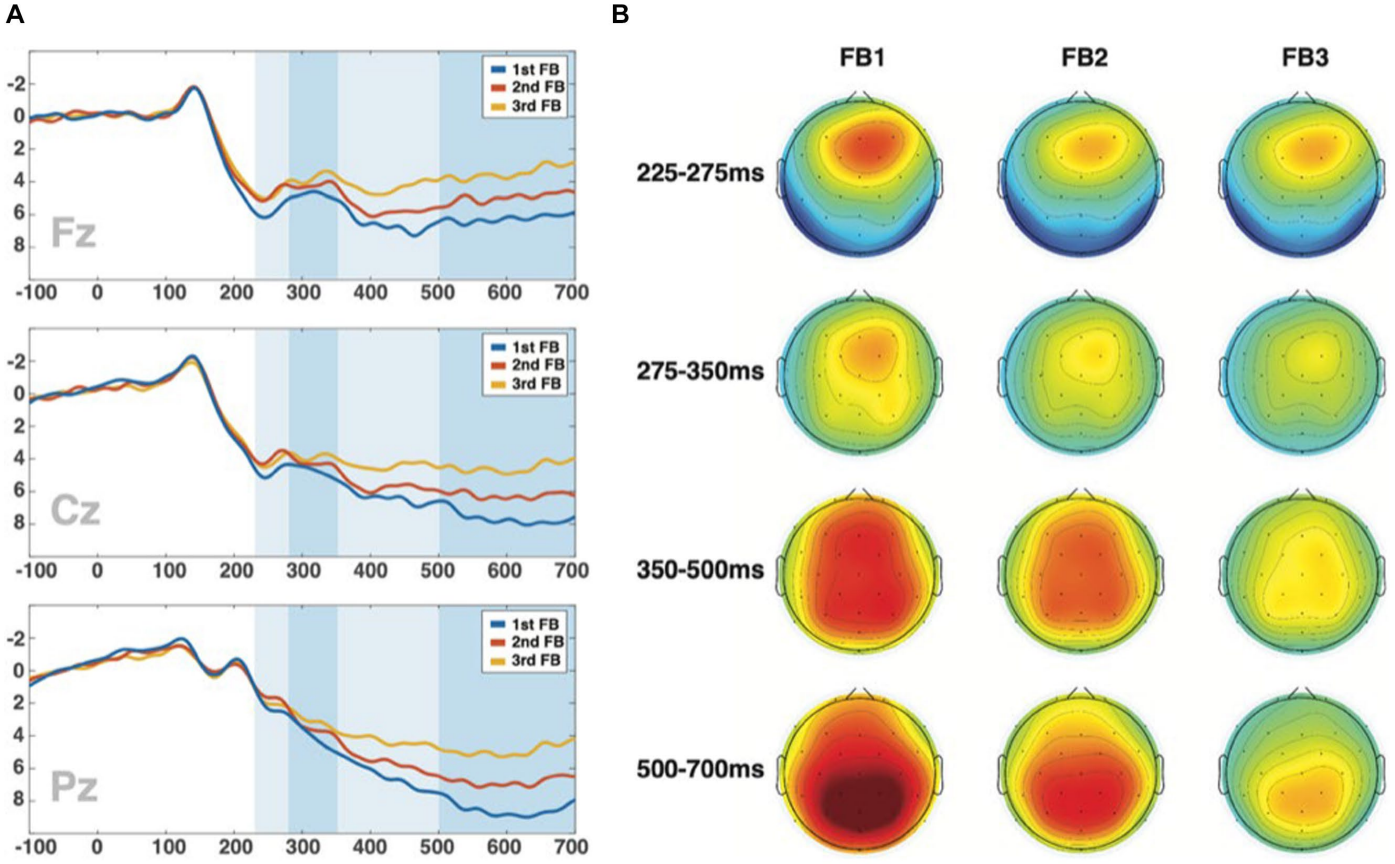
**(A)** ERPs at the central electrodes (Fz, Cz, Pz) for every feedback and the identification of the different ranges of interest over signals (225–275 ms., 275–350 ms., 350–500 ms., 500–700 ms.). **(B)** Topographies at three feedback conditions through the intervals.

Figure 4 shows the average ERPs for the three FB presentations at the Fz, Cz, and Pz electrodes (Figure 4A) and their topographic representations (Figure 4B). Figure 4A reveals that all electrodes presented an amplitude reduction with every feedback repetition. We analyzed four different time ranges (225 to 275 ms, 275–350 ms, 350 to 500 ms, and from 500 to 700 ms) corresponding to the different components found in the ERPs.

The topographical maps of the four studied time ranges (Figure 4B) showed a clear frontocentral activity in the first interval (225–275 ms) and second interval (275–350 ms), more centroparietal at 350–500 ms and clearly posterior at 500–700 ms, which is reduced in the second and third FB compared to the first one. Consistently, BMM revealed this signal reduction in the first adjustment (FB1-FB2) in frontal electrode with robust credible evidence (±0.05*SD*y* ROPE) at earliest interval (HDI(95%): FB1-FB2: 225–275 ms: Fz: [−1.09 – −0.56]), and then in the last interval (HDI(95%): FB1-FB2: 500–700 ms: Fz: [−1.12 – −0.57]; Cz: [−1.16 – −0.59]; Pz: [−1.31 – −0.63]). Then, in the second adjustment (FB2-FB3), we found signal differences in all electrodes in the third and last intervals (HDI(95%): FB2-FB3: 350–500 ms: Fz: [−1.81 – −0.97]; Cz: [−1.58 – −0.74]; Pz: [−1.31 – −0.63]; FB3-FB2: 500–700 ms: Fz: [−1.98 – −1.13]; Cz: [−2.19 – −1.34]; Pz: [−2.50 – −1.63]). The variability of this model, used for ROPE, ranged from *SDy_min_* = 9.50 to *SDy_max_* = 11.87.

#### 3.2.2 Discrepancy × feedback model

**Figure 5:**
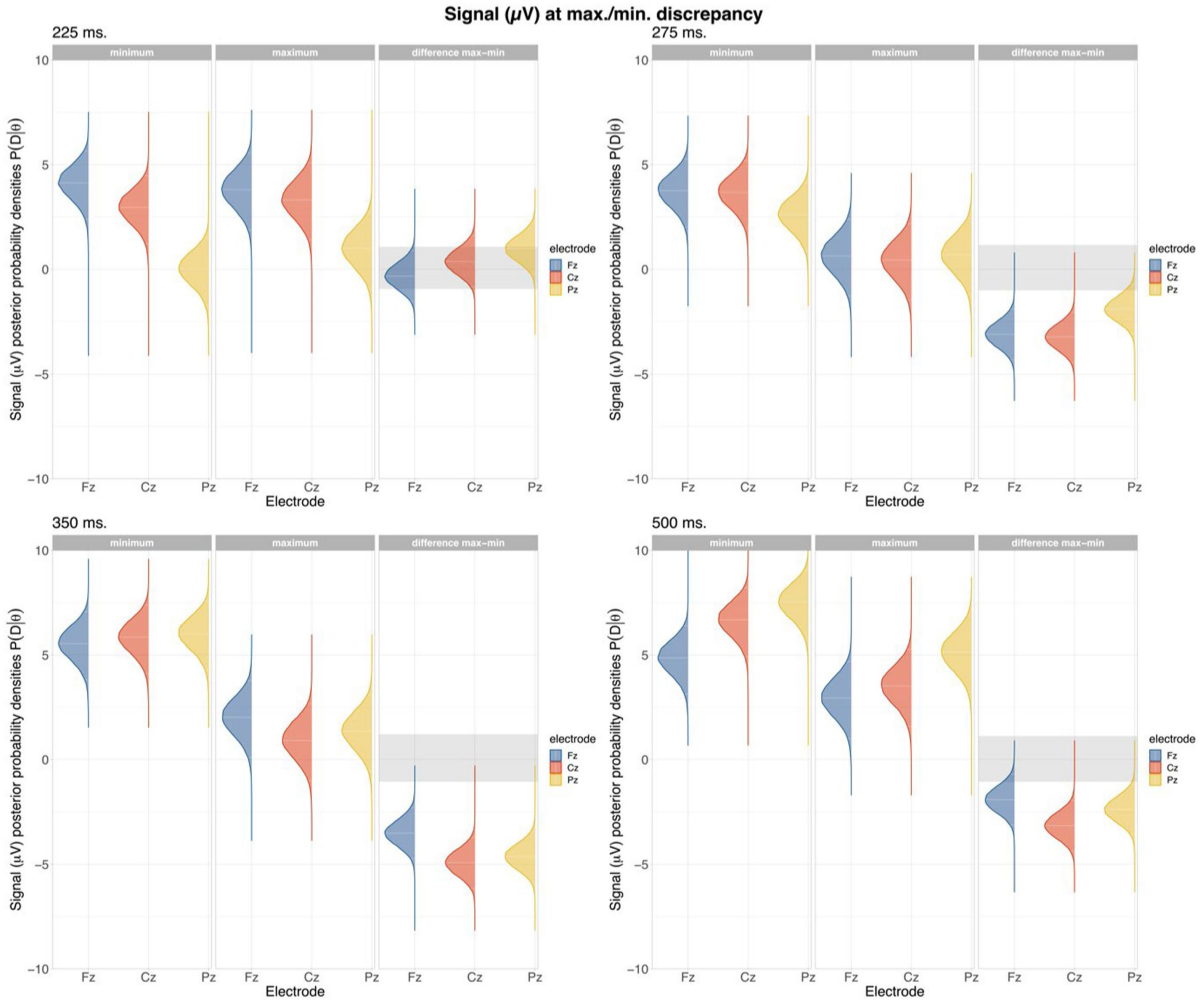
Density plots showing minimum, maximum and differences in signal from the posterior data ( $P\left(D|\theta \right)$ ) at maximum Discrepancy in 1st feedback. Shadowed area in the differences plot represents the empirical ROPE. Dimmed white lines inside the posterior density represent three percentiles (0.025, 0.5, 0.975).

The next model was the Discrepancy-FB interaction model, which predicted differences in signal regarding the maximum and minimum discrepancy at every FB (Figure 5). As we expected, we found credible evidence of differences in signal between the highest and lowest degree of discrepancy in partners’ responses in the first two feedbacks. There was a credible decreased signal in maximum discrepancy in all electrodes starting at the second interval (HDI(95%): FB1: 275–350 ms: Fz: [−4.54 – −2.51]; Cz: [−5.09 – −3.05]; Pz: [−4.13 – −2.09]), and also in the third interval (HDI(95%): FB1: 350–500 ms: Fz: [−4.38 – −2.28]; Cz: [−6.05 – −3.97]; Pz: [−5.92 – −3.89]), that shifted to only parietocentral in the last interval (HDI(95%): FB1: 500–700 ms: Cz: [−3.93 – −1.85]; Pz: [−3.25 – −1.21]). Consistently, we found similar results in FB2, starting from a general decrease in the three electrodes in the second and third intervals (HDI(95%): FB2: 275–350 ms: Fz: [−3.80 – −1.49]; Cz: [−4.56 – −2.28]; Pz: [−3.70 – −1.45]; 350–500 ms: Fz: [−3.59 – −1.24]; Cz: [−5.45 – −3.15]; Pz: [−5.46 – −3.22]), which shifted to only parietocentral in the last interval (HDI (95%): FB2: 500–700 ms: Cz: [−3.62 – −1.30]; Pz: [−3.25 – −0.96]). Finally, in the last feedback, where the divergences were lower, there was credible evidence of signal decrease related to the degree of divergence only in second interval and central area (HDI(95%): FB3: 275–350 ms: Cz: [−3.70 – −0.62]) The variability of this model ranged from *SDy_min_* = 9.50 to *SDy_max_* = 11.87.

#### 3.2.3 Adjustment analysis

Contrary to our initial hypothesis, we found no credible evidence using the adjustment as a predictor.

### 3.3 Time-frequency analysis

#### 3.3.1 Feedback model

**Figure 6:**
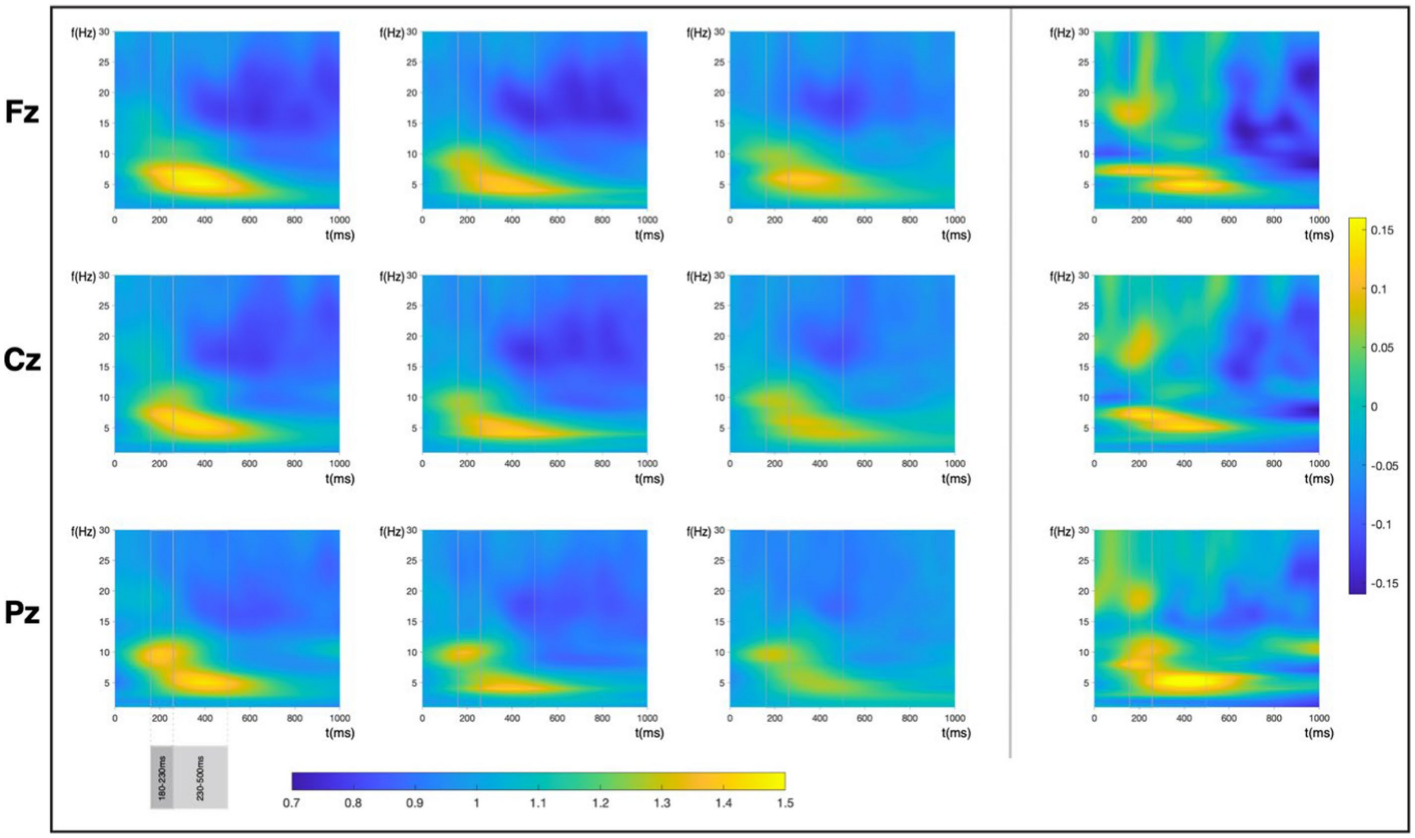
Time-frequency plots depicting the three feedback power changes per electrode and the difference between the 1st and the 3rd feedback power.

Figure 6 shows the time-frequency results for the three studied electrodes and the three feedbacks. Note that every time interval has its own model (to increase computational simplicity and efficiency). Therefore, we will intentionally inform about model variability structures in Table 3 to keep clarity. We analyzed two different time ranges, early (180 to 230 ms), and late (230 to 500 ms). Results showed an enhancement of theta activity in the first FB with an evident decrease at every FB repetition. The BMM revealed consistent and credible evidence (±0.01*SD*y* ROPE) for this reduction in the first studied time range (180–230 ms) only for the second adjustment and in Pz (HDI(95%): FB3-FB2: 180–230 ms: Pz: [−0.070 – −0.016]). In the next time range (230–500 ms), we find decrease of power in the first adjustment for all three electrodes (HDI(95%): FB2-FB1: 230–500 ms: Fz: [−0.075 – −0.030]; Cz: [−0.070 – −0.024]; Pz: [−0.075–0.029]), but not credible differences in theta in this time range in the second adjustment.

**Table 3:** Variability of different time-frequency models in the simple feedback model.

Frequency	Time-Interval	SDymin	SDymax
Theta	180–230 ms	0.91	1.03
Theta	230–500 ms	0.88	1.03
Alpha	180–230 ms	1.04	1.33
Alpha	230–500 ms	0.85	1.03
Beta	180–230 ms	0.67	0.87
Beta	230–500 ms	0.48	0.56

Alpha activity showed no credible differences in the first studied time range. In contrast, in the second interval (230–500 ms), there was a consistent reduction in the alpha band in the three electrodes in the first adjustment (HDI(95%): FB2-FB1: 230–500 ms: Fz: [−0.069 – −0.013]; Cz: [−0.095 – −0.038]; Pz: [−0.083 – −0.030]) that shifted to a centroparietal increase in the second adjustment (HDI(95%): FB3-FB2: 230–500 ms: Cz: [0.035–0.095]; Pz: [0.017–0.075]).

Finally, regarding beta activity changes throughout the trial, we found credible evidence in the first interval (180–230 ms) of a decreased activity in the first adjustment (FB2-FB1) in the three electrodes (HDI(95%): Fz: [−0.069 – −0.022]; Cz: [−0.062 – −0.016]; Pz: [−0.061 – −0.014]). However, this decrease was neither sustained in the next interval nor in the second adjustment.

#### 3.3.2 Discrepancy × feedback model

In the discrepancy and feedback interaction model, we found changes associated with the highest degree of discrepancy only in FB1 for alpha and beta bands and in the three electrodes in the second time interval (HDI (95%), Alpha: 230–500 ms: Fz: [−0.692 – −0.175]; Cz: [−0.729 – −0.208]; Pz: [−0.701 – −0.184]); Beta: 230–500 ms: Fz: [−0.536 – −0.169]; Cz: [−0.520 – −0.156]; Pz: [−0.543 – −0.178] when the discrepancy was at the highest point.

#### 3.3.3 Adjustment × feedback model

Finally, in the adjustment interaction model, we found power change associated with the highest degree of adjustment only in the theta frequency band in FB2-FB1. Our results indicated credible evidence of a change in theta power in the second time interval and in parietal electrode (HDI(95%):230–500 ms: Pz: [0.015–0.150]).

## 4 Discussion

In the present paper, we used a new experimental design to study the neurophysiological mechanisms of social conformity. Results showed, first, that participants tended to converge in their decisions even when this was neither explicitly stated nor rewarded, a tendency that was consistent in both studies. We also showed evidence in Study 1 that cooperation credibly shifts this tendency to the pursuit of convergence at the end of the trial. Moreover, cooperation enhanced a more synchronic, trusting, and rewarding subjective experience, translating into a convergence-seeking behavioral adaptation. Importantly, these results showed that previous collaboration activated psychological predispositions towards seeking social rewards such as convergence even when, according to our evidence, this does not necessarily mean getting better accuracy in their results.

Second, we showed that ERPs signaled specific differences related to the degree of discrepancy between participants. According to results in the first feedback, the voltage in the second interval (275–350 ms) was more negative when the discrepancy between the two participants was higher than when it was lower, especially at frontocentral electrodes. This is compatible with the Feedback-Related Negativity ERP, that appears after negative feedback (Miltner et al., 1997) and is modulated by prediction error (Sambrook and Goslin, 2015). It is essential to note that, although the spatial distribution of this component is compatible with the FRN (with differences at Fz and Cz, but not at Pz, see Figure 5), its latency is delayed than the classical described for FRN (250–300 ms after feedback presentation, Falkenstein et al., 1990; Marco-Pallarés et al., 2008; Sambrook and Goslin, 2015). This delay could be related to the higher complexity of the feedback in our study (two numbers that have to be contrasted) compared with the much simpler stimuli used in most studies describing this component (i.e., images indicating positive or negative feedback). Therefore, given that the discrepancy in this model shows the difference in signal related to the maximum discrepancy in responses between dyads, an increase in the negativity of this component associated with high discrepancy trials could indicate “worse than expected” agreement in the initial estimation of the two participants (higher prediction error), yielding a higher FRN. However, our results contradict previous evidence (Pierguidi et al., 2019) that related important negativity around the 200 ms (N2) related to conformity as we did not find such clear negativity in signal in our data, and the credible differences happened after the second interval, from 275 ms on. Nonetheless, other recent findings in ERP conformity studies (Bogdan et al., 2022) found significant differences not in the N2 but in the following positivity. This discrepancy could arise from the differences in the experimental tasks used in the different studies. In our and Bogdan et al. (2022) experiments, participants were asked to make a complex decision-making process associated with the will to conform based on prediction and learning. In our case, a participant’s decision to converge is based not only on how close or far their responses are to the target but also on their willingness to make them match the other partner. Hence, while our and Bogdan et al. (2022) proposal is dependent on different regimes of expectations, the task proposed by Pierguidi et al. (2019) focused on word conflict and norm deviation, fundamentally linked to conflict processing and, hence, its related responses.

Contrary to our expectation, we did not find credible evidence relating ERPs with adjustment as suggested by previous studies (Pisauro et al., 2017; Herding et al., 2019; Desender et al., 2021; Bogdan et al., 2022). However, results showed the involvement of theta oscillatory activity with adjustment in the first feedback. Theta activity has consistently been associated with cognitive conflict, prediction error, and surprise, among many other functions (see Cavanagh et al., 2012, for a review), with its main generators located in the medial prefrontal Cortex (Mas-Herrero and Marco-Pallarés, 2016). In addition, it has been proposed that this component plays a vital role in the top-down cognitive control necessary for the behavioral and strategic adjustment essential in the decision-making process after an unexpected result (Cavanagh et al., 2010) or the adaptive control under uncertainty (Cavanagh et al., 2012), which would be compatible to a role in adjustment, especially in those situations in which it is more critical (that is, the first adjustment).

Besides, in the simple feedback change model, Alpha activity showed a different pattern, with a decrease in power in the first adjustment (FB2-FB1) and an increase in the second. The role of alpha in cognitive control functions has been described as a signal to alertness (see Sadaghiani and Kleinschmidt, 2016, for a review). The role of alpha as a top-down physiological inhibitor has also been studied in non-human animal studies suggesting alpha oscillations increase when neuronal activity of the brain region decreases (Haegens et al., 2011). Our results would suggest a certain coherence to this interpretation, as attentional engagement is still required, or even required to be enhanced, in the first adjustment. In contrast, these requirements drop in the second as participants are closer to their goal.

Finally, results in the beta band suggest an early activity decrease in the first adjustment in the three electrodes. Additionally, *Discrepancy* model relates to evidence of frontocentral power decrease in beta frequency. Recent evidence with conforming decision-making (Wang et al., 2020) suggests beta be sensitive to correct outcome evaluation; therefore, applying these results to our paradigm, we would expect higher beta power when convergence was higher. Our results could be consistent with this claim, which would be in line with the proposed role of beta oscillations in reward processing (Mas-Herrero et al., 2015), acting as a motivational signal that could mediate different cognitive processes (see Marco-Pallarés et al., 2015 for a review). Therefore, convergence could act as an intrinsic reinforcer, as suggested by Study 1, increasing beta power when the discrepancy between the estimations of the two participants was lower. Alternatively, prefrontal beta activity has also been proposed to be a mechanism for flexible control and allocation of working memory (Lundqvist et al., 2018; Schmidt et al., 2019) with a reduction during the encoding of relevant information (Schmidt et al., 2019). This could be compatible with the reduction of beta activity in those trials with the higher discrepancy, which were those that required a higher change to reach the convergence. In addition, present results would also support the proposed role of beta oscillatory activity in signaling the status quo (Engel and Fries, 2010). According to this proposal, beta activity would be higher in those cases in which the current state will be maintained compared to those in which a change is predicted. Therefore, in our results, beta activity would be higher in those trials showing lower discrepancy (higher convergence), as they would require no (or more minor) change than those with higher discrepancy, which would probably require an adjustment in future. Given that these alternative accounts could explain our results, future studies manipulating attentional demands of similar conformity tasks could help interpret an in-depth functional role of these oscillatory components.

The current study also offers some interesting insights into the role of emotions and affective processing in social decision-making, particularly in behavioral adaptation associated with conformity. First, the results of Study 1 clearly showed that cooperation prior to the main experiment enhanced convergence between dyads, as well as their enjoyment of the task (likeability) and the reward of working with their peers. This is in line with the idea that humans are intrinsically and strongly motivated to collaborate with others in contrast with, e.g., other primates, Tomassello (2023) and that positive emotions are related to cooperation and prosocial behavior (Rand et al., 2015) and might increase conformity (Heerdink et al., 2013). Therefore, cooperating in the pre-task before the main experiment might enhance the dyad’s affiliation and their shared positive emotions, yielding to a greater willingness to converge in the task (Cialdini and Goldstein, 2004). In addition, an essential result of the present experiment is that the behavioral adjustment to conform to their peers appeared spontaneously in all the groups of Study 1 and 2 without any explicit instruction or incentive to do so. This convergence-seeking could arise from the positive emotions associated with it (Cialdini and Goldstein, 2004) and the avoidance of negative emotions associated with conflict (Klucharev et al., 2009).

Interestingly, a recent study has proposed that social behavior is driven by reward prediction errors and emotional prediction errors (Heffner et al., 2021). By modelling prediction errors of explicit rewards and emotional components (valence and arousal), the authors found that emotion prediction errors played a crucial role in social decisions, even stronger than reward prediction errors. As stated above, in the main task of current experiments, there are no explicit rewards that might induce reward prediction errors, but some of the ERP and oscillatory components found (in particular, FRN, theta and beta activities, Marco-Pallarés et al., 2008, 2015; Mas-Herrero et al., 2015, Mas-Herrero and Marco-Pallarés, 2016) have been related to the prediction error processing. Therefore, it might be the case that these prediction error signals were raised not by rewarding stimuli but by discrepancies in the expected emotional valence of the trials. Therefore, high discrepancies in the estimations of the dyads and the difficulty in converging could induce negative emotional valence prediction errors. In contrast, successful trials (e.g., the same estimation in the first feedback) could yield to positive valence prediction errors. These prediction errors could be related to the components mentioned above without the need for an explicit reward. Additionally, anticipatory emotions could also play an important role in the present experiment. These emotions are generated by the prospect and the uncertainty and risks of future outcomes (Loewenstein et al., 2001; Wang et al., 2022). In the present experiment, outcomes in a trial might indicate the behavior of participants in the future. Therefore, for example, a trial in which two participants have changed their estimations to reach convergence might be indicative of the willingness of the dyad to reach convergence in the future, yielding to positive anticipatory emotions. In contrast, a high divergence in the first repetition of a trial with no change of estimation in the next repetitions might show difficulties in convergence in future trials. However, it is important to note that in the current experimental paradigm anticipatory emotions cannot be clearly dissociated from emotions generated by the outcome of the trial. Future studies with direct manipulations of emotional valence and arousal could help disentangle the role of emotional prediction error and anticipatory emotions in the neurophysiological responses associated with behavioral social adjustment.

Our study, as any other study, is not absent of limitations. Firstly, even if the task design was purposefully unaltered as we wanted to capture the phenomena unbiased, it is also true that the lack of manipulations limited our interpretation. In this sense, the inclusion in Study 2 of a condition with less convergence (e.g., a group explicitly encouraging the convergence or the individual group in Study 1) could help dissociate those brain responses associated with social adjustment from those related to more domain-general processes. Secondly, we acknowledge that the number of dyads in our study is not very large. Nonetheless, using the Bayesian framework and its avoidance of asymptotic limits is helpful in this specific limitation.

To summarize, the present results support the idea that the proposed paradigm is valid to study the neural correlates of convergence mechanisms and goes beyond previous experimental paradigms that have focused on segmented parts of the conformity process, allowing the study of this phenomenon in a more holistic way. Future directions on this task imply the verification and extension of the cognitive processes found in current research and the modulation of these processes manipulating different conditions, such as threat, social categorization, polarization processes, etc. In addition, future implementations of the task could involve increasing the number of people working together, a fine-grain control of the intimacy levels of the dyads, or the study of different populations with neuropsychiatric conditions affecting social cognition.

## Data availability statement

The datasets presented in this study can be found in online repositories. The names of the repository/repositories and accession number(s) can be found at: https://www.openicpsr.org/openicpsr/project/192864/version/V2/view.

## Ethics statement

The studies involving humans were approved by Bioethics Committee of University of Barcelona. The studies were conducted in accordance with the local legislation and institutional requirements. The participants provided their written informed consent to participate in this study.

## Author contributions

UV: Conceptualization, Data curation, Formal analysis, Investigation, Methodology, Project administration, Writing – original draft, Writing – review & editing. AA: Methodology, Writing – review & editing. MP-L: Resources, Supervision, Writing – review & editing. JM-P: Conceptualization, Funding acquisition, Resources, Supervision, Validation, Writing – review & editing.
